# Gene, Stem Cell, and Alternative Therapies for SCA 1

**DOI:** 10.3389/fnmol.2016.00067

**Published:** 2016-08-12

**Authors:** Jacob L. Wagner, Deirdre M. O'Connor, Anthony Donsante, Nicholas M. Boulis

**Affiliations:** Boulis Laboratory, Department of Neurosurgery, Emory School of Medicine Atlanta, GA, USA

**Keywords:** SCA 1, gene therapy, stem cell therapy, mouse model, Ataxin-1, RNAi

## Abstract

Spinocerebellar ataxia 1 is an autosomal dominant disease characterized by neurodegeneration and motor dysfunction. In disease pathogenesis, polyglutamine expansion within Ataxin-1, a gene involved in transcriptional repression, causes protein nuclear inclusions to form. Most notably, neuronal dysfunction presents in Purkinje cells. However, the effect of mutant Ataxin-1 is not entirely understood. Two mouse models are employed to represent spinocerebellar ataxia 1, a B05 transgenic model that specifically expresses mutant Ataxin-1 in Purkinje cells, and a *Sca1 154Q/2Q* model that inserts the polyglutamine expansion into the mouse Ataxin-1 locus so that the mutant Ataxin-1 is expressed in all cells that express Ataxin-1. This review aims to summarize and evaluate the wide variety of therapies proposed for spinocerebellar ataxia 1, specifically gene and stem cell therapies.

## Introduction

Spinocerebellar ataxia (SCA) is a group of autosomal dominant neurodegenerative diseases characterized by progressive degeneration in the spinal cord, brain stem, and cerebellum. SCA types 1–36 have been identified, each attributable to a different gene. SCA genetic abnormalities are most commonly caused by cytosine-adenosine-guanine (CAG) repeat expansion leading to a polyglutamine expanded protein product presumed to be toxic to neurons (Whaley et al., 2011; Table 1).

**Table 1:** **Autosomal dominant hereditary ataxias**.

**Disease Name^a^**	**Gene**	**Average onset (range in years)**	**Average duration (range in years)**	**Distinguishing features^b^**
SCA1	*ATXN1*	3rd–4th decade (< 10 to >60)	15 years (10–28)	Pyramidal signs, peripheral neuropathy
SCA2	*ATXN2*	3rd–4th decade (< 10 to >60)	10 years (1–30)	Slow saccadic eye movements, peripheral neuropathy, decreased DTRs, dementia
SCA3	*ATXN3*	4th decade (10–70)	10 years (1–20)	Pyramidal and extrapyramidal signs; lid retraction, nystagmus, decreased saccade velocity; amyotrophy fasciculations, sensory loss
SCA4	16q22.1	4th–7th decade (19–72)	Decades	Sensory axonal neuropathy, deafness; may be allelic with 16q22- linked SCA
SCA5	*SPTBN2*	3rd–4th decade (10–68)	>25 years	Early onset, slow course; first reported in descendants of Abraham Lincoln
SCA6	*CACNA1A*	5th–6th decade (19–71)	>25 years	Sometimes episodic ataxia, very slow progression
SCA7	*ATXN7*	3rd–4th decade (0.5–60)	20 years (1–45; early onset correlates with shorter duration)	Visual loss with retinopathy
SCA8	*ATXN8I*	4th decade (1–65)	Normal life span	Slowly progressive, sometimes brisk DTRs, decreased vibration sense; rarely, cognitive impairment
	*ATXN80S*			
SCA10	*ATXN10*	4th decade (12–48)	9 years	Occasional seizures; most families are of Native American background
SCA11	*TTBK2*	Age 30 years (15–70)	Normal life span	Mild, remain ambulatory
SCA12	*PPP2R2B*	4th decade (8–62)		Slowly progressive ataxia; action tremor in the 30s; hyperreflexia; subtle Parkinsomism possible; cognitive/psychiatric disorders including dementia
SCA13	*KCNC3*	Childhood or adulthood	Unknown	Mild intellectual disability, short stature
SCA14	*PRKCG*	3rd–4th decade (3–70)	Decades (1–30)	Early axial myoclonus
SCA15	ITPR1	4th decade (7–66)	Decades	Pure ataxia, very slow progression
SCA16	*SCA16*	Age 39 years (20–66)	1–40 years	Head tremor; one Japanese family
SCA17	*TBP*	4th decade (3–55)	>8 years	Mental deterioration; occasional chorea, dystonia, myoclonus
SCA18	7q22-q32	Adolescence (12–25)	Decades	Ataxia with early sensory/motor neuropathy, nystagmus, dysarthria, decreased tendon reflexes, muscle weakness, atrophy, fasiculations, Babinski responses
SCA 19/22	*KCND3*	4th decade (10–51)	Decades	Slowly progressive, rare cognitive impairment, myoclonus, hyperreflexia
SCA20	11q12.2	5th decade (19–64)	Decades	Early dysarthria, spasmodic dysphonia, hyperreflexia, bradykinesia; calcification of the dentate nucleus
	11q12.3			
SCA21	*SCA21*	6–30	Decades	Mild cognitive impairment
SCA23	*PDYN*	5th–6th decade	>10 years	Dysarthria, abnormal eye movements, reduced vibration and position sense; one Dutch family; neuropathology
SCA25	*SCA25*	(1.5–39)	Unknown	Sensory neuropathy; one French family
SCA26	*EEF2*	(26–60)	Unknown	Dysarthria, irregular visual pursuits; one Norwegian-American family; MRI: cerebellar atrophy
SCA27	*FGF14*	Age 11 years (7–20)	Decades	Early-onset tremor; dyskinesia, cognitive deficits; one Dutch family
SCA28	*AFG3L2*	Age 19.5 years (12–36)	Decades	Nystagmus, opthalmoparesis, ptosis, increased tendon reflexes; two Italian families
SCA29	3p26	Early childhood	Lifelong	Learning deficits
SCA30	4q34.3–q35.1	(45–76)	Lifelong	Hyperreflexia
SCA31	*BEAN1*	5th–6th decade	Lifelong	Normal sensation
SCA35	*TGM6*	43.7 ± 2.9 (40–48) years	15.9 ± 8.8 (5–31) years	Hyperreflexia, Babinski respsonses; spasmodic torticollis
SCA36	*NOP56*	52.8 ± 4.3 years	Decades	Muscle fasiculations, tongue atrophy, hyperreflexia

*DTR, deep tendon reflex*.

a *SCA9 has not been assigned*.

b *All have gait ataxia*.

*Reprinted by permission from Macmillan Publishers Ltd: Genetics in Medicine (Jayadev and Bird, 2013), copyright (2013)*.

In spinocerebellar ataxia 1 (SCA 1), the accumulation of the mutant ataxin-1 protein (ATXN1) causes loss of cerebellar Purkinje cells (PC) and dysfunction and degeneration in the cerebellum, brain stem, and spinal cord. To date, treatment consists of managing the symptoms with pharmacologic agents. No fundamental therapies for SCA 1 have been identified yet (Whaley et al., 2011), although several experimental studies have shown promising results.

This review will provide background information on SCA 1 and the mouse models used as well as inform the reader about promising gene and stem cell therapy approaches. Challenges regarding these novel approaches will be discussed and other alternative therapies will be briefly reviewed.

## Facts and demographics

According to the National Ataxia Foundation, approximately 1–2 in 100,000 people will develop SCA 1, but the frequency varies depending on ethnic background and location (National Ataxia Foundation, https://www.ataxia.org/).

The onset of the disease begins around the 3rd–4th decade but can develop between the ages of 4 and 74 (Schols et al., 2004). From onset, patient survival ranges from 10 to 28 years (average = 15 years; Jayadev and Bird, 2013). In cases of early onset (before 13 years old), disease progression is more rapid and severe, with patients usually dying before the age of 16 (Zoghbi et al., 1988).

Clinically, SCA 1 symptoms include limb ataxia, gait disturbance, slurred speech, balance difficulty, brisk tendon reflexes, hypermetric saccades, nystagmus, mild dysphagia, and cognitive impairment. SCA 1 symptoms can be distinguished from other hereditary ataxias by the predominance of pyramidal symptoms. Amyotrophy and sensory loss can also be present in affected individuals. Olivopontocerebellar atrophy is the major finding on MRI/CT (Schols et al., 2004). Eventually, affected individuals develop respiratory failure, the main cause of death for SCA 1 (Subramony and Ashizawa, 1993).

## Molecular pathogenesis

SCA 1 is caused by a CAG repeat expansion in the *ATXN1* gene (OMIM: 601556) that encodes ATXN1. In normal *ATXN1*, the triplet CAG is repeated 6 to 36 times while in mutant forms (polyQ-ATXN1) the number of repeats can exceed 100. Similar to other polyglutamine (polyQ) diseases such as Huntington's, as the polyQ expansion becomes longer, disease onset occurs earlier, and the symptoms are more severe (Kang and Hong, 2009). The polyQ expansion is thought to be due to errors in the DNA replication machinery (Kang et al., 1995). The number of repeats can increase from generation to generation, particularly in the paternal germline (Matilla et al., 1993).

ATXN1 is mainly localized to the nucleus of neuronal cells; however, in Purkinje cells, it is found in both the cytoplasm and the nucleus (Servadio et al., 1995). It associates with a collection of high molecular weight protein complexes that also contain Capicua (CIC), a transcriptional repressor containing a high mobility group (HMG) box. The presence of human polyQ-ATXN1 alters the size distribution of these complexes and inhibits the repressor activity of CIC. Overexpression of CIC in a fly model suppresses the morphologic changed induced by polyQ-ATXN1 (Lam et al., 2006). Additionally, Yue et al. found that ATXN1 binds RNA and that, as size of its polyQ tract increases, its ability to bind RNA decreases. Thus, expansion of the ATXN1 polyQ tract may alter its role in RNA metabolism (Yue et al., 2001). polyQ-ATXN1 aggregates and forms inclusions in the nucleus and cytoplasm of neurons, especially in PCs (Kang and Hong, 2009). PolyQ-ATXN1 alters several cellular pathways, including transcription (Cvetanovic et al., 2007; Lee S. et al., 2008), RNA processing (Hong et al., 2003), and signal transduction (Goold et al., 2007; Gatchel et al., 2008), disrupting normal cell function and leading to cell death (Kang and Hong, 2009).

Phosphorylation contributes to the regulation of ATXN1 folding and distribution. Emamian et al. found that serine 776 (S776) was phosphorylated in transgenic mice carrying ATXN1. Mutating S776 to alanine altered the intracellular deposition of polyQ-ATXN1, preventing nuclear inclusions from forming. When the S776 and A776 forms of polyQ-ATXN1 (polyQ-ATXN1-S776 and polyQ-ATXN1-A776, respectively) were expressed in Purkinje cells in mice, polyQ-ATXN1-A776 was substantially less toxic than polyQ-ATXN1-S776, demonstrating that phosphorylation of this serine residue contributes significantly to the harmful effects of the mutant protein (Emamian et al., 2003). The protective effect of A776 appears to be due to the fact that polyQ-ATXN1-A776 does not produce the abnormal, high molecular weight complexes that are observed in mice carrying polyQ-ATXN1-S776, even though both forms associated strongly with CIC. Interestingly, it appears that toxicity arises, not due to novel interactions between polyQ-ATXN1 and other proteins, but from polyQ-ATXN1 adversely affecting the function of proteins it normally interacts with (Lam et al., 2006). Protein Kinase A (PKA) is the cAMP-dependent kinase responsible for S776 of ATXN1 (Chen et al., 2003; Jorgensen et al., 2009). In addition to S776, Vierra-Green et al. found through matrix-assisted laser description ionization time-of-flight mass spectrometry (MALDI TOF) and mutational analysis that S239 is also a phosphorylation site of ATXN1 (Vierra-Green et al., 2005), although further research is required to determine if S239 is linked to SCA 1 pathogenesis. Understanding the phosphorylation of ATXN1 allows for a better understanding of the role ATXN1 has within neurons, specifically with post-translational modifications, which is vital for future therapeutic strategies.

Relevant to polyQ-ATXN1 cytotoxicity, Lee et al. found that ATXN1 levels might be post-transcriptionally regulated by miRNA, specifically miR-19, miR-101, and miR-130. When miR-19, miR101, and miR130 were transfected into HEK293T, HeLa and MCF7 cells, a marked decrease in ATXN1 levels was observed. When 2′-O-methyl inhibitors against the miRNA were added to HEK293T cells, ATXN1 levels increased. These findings present the possibility that miRNA-binding site mutations or miRNA genes might lead to the SCA 1 neurodegenerative phenotypes (Lee Y. et al., 2008).

## Mouse models

Mouse models have been generated to study the cerebellar degeneration associated with SCA 1. In the B05 transgenic mouse model (B05) the human SCA 1 cDNA was modified to contain 82 CAG repeats and expressed under the control of the Purkinje cell protein 2 (pcp2) promoter to form a pcp2/SCA1 transgene. The 5′ regulatory sequences of *pcp2* are sufficient to restrict transgene expression to PCs (Oberdick et al., 1990; Vandaele et al., 1991). B05 transgenic mice overexpress polyQ-ATXN1, which leads to the loss of PCs from the cerebellum and thus present the neurological phenotype of ataxia. Abnormalities include reduced activity, head swaying and incoordination. Ataxia onset begins at 12 weeks of life. However, because the mutant cDNA is only expressed in Purkinje cells, this model only displays symptoms of degenerating PCs and these mice have a normal life span (Burright et al., 1995). Patients, in contrast, experience dysfunction in a variety of neurons in addition to PCs (Bürk et al., 2001). These other neurons produce cognitive dysfunction that is not represented in the B05 mouse model (Table 2), which makes comparisons to SCA 1 patients difficult.

**Table 2:** **Overview of mouse models for SCA 1**.

	**B05 transgenic mice (B05)**	***Sca1 154Q/2Q* mice (154Q/2Q)**
Phenotype	Motor incoordination, ataxia (12 weeks), no cognitive impairment	motor incoordination, muscle wasting, cognitive impairment, memory deficits
Physical Onset	5 weeks	7–8 weeks
Course of the disease	Normal life span	Premature, 35–45 weeks
Neuropathology	PC loss, Bergmann glial proliferation, shrinkage and gliosis of molecular layer	Reduced dendritic arborization of PCs (early stage)
		Neuronal intranuclear inclusions and PC loss (advanced disease)
	Purkinje neuron dendritic and somatic atrophy	Hypocampal synaptic dysfunction but no significant loss
Mechanism	Overexpression of polyQ-ATXN1 (82 CAG repeats) under the control of Purkinje Cell pcp2 promoter	Expanded repeat of 154 CAGs was inserted into the mouse Sca 1 locus (knock-in)
References	Burright et al., 1995; Clark et al., 1997	Watase et al., 2002

To better model SCA 1, Watase et al. generated a SCA 1 knock-in model by introducing a 154-CAG expansion into exon 8 of the endogenous ATXN 1 locus. The knock-in mouse model, called *Sca1 154Q/2Q* (154Q/2Q), expresses polyQ-ATXN1 in neurons in the cortex, thalamus, hypocampus, caudate nucleus, putamen, brain stem, cerebellum, and spinal cord. With polyQ-ATXN1 allele present throughout the central nervous system, the 154Q/2Q model displays more of the human disease features, such as motor incoordination, muscle wasting, cognitive impairment, and premature death, making it a more relevant physiological model of the disease. Disease onset begins at 7–8 weeks and mice die prematurely at 35–45 weeks of age (Watase et al., 2002; Table 2).

No non-rodent animal model exists for SCA 1. A transgenic model of Huntington's disease, another polyQ disease involving an expanded polyglutamine repeat in *HTT*, exists in rhesus macaque (Yang et al., 2008), suggesting that a SCA 1 model could be made as well.

## Gene therapy

SCA 1 is a monogenic disease; thus, gene therapy is an obvious treatment approach. Gene therapeutic intervention involves either reducing expression of the mutant *ATXN1* through gene silencing or overexpressing a paralog of ATXN1, ataxin-1-like (ATXN1L), to competitively inhibit the formation of toxic complexes by polyQ-ATXN1 (Keiser et al., 2013).

The RNAi approach regulates gene expression through small RNAs, including artificial microRNA (miRNA), small interfering RNA (siRNA), and short hairpin RNA (shRNA). Short hairpin RNA, siRNA, and miRNA are all non-coding RNA that post-transcriptionally regulate mRNA transcript levels. siRNA acts on a particular sequence for degradation while miRNA is more likely to target mRNA for transcriptional silencing (Lam et al., 2015). Unlike siRNA which requires two independent RNA strands, a sense and antisense strand that guides the RNA-induced silencing complex (RISC), shRNA is a single strand that folds back on itself to produce a double-stranded molecule (Davidson and Paulson, 2004). The Davidson research group showed that RNAi platforms (Keiser et al., 2013) expressed using viral vectors could potentially have therapeutic effects on polyglutamine diseases. Xia et al. investigated the possibility that virally expressed siRNA could decrease levels of polyglutamine expanded proteins in neural PC12 clonal cells. The lines were developed to express tetracycline-repressible eGFP-polyglutamine fusion proteins, which the siRNA targeted. Decrease in protein aggregate levels was shown by western blot analysis and cellular fluorescence (Xia et al., 2002; Table 3).

**Table 3:** **Overview of gene therapies for SCA 1**.

	**Treatment/vector**	**Animal model**	**Route**	**Outcome**
Keiser et al., 2013	(1) miRNA/AAV serotype 2/1	B05 mice	DCN injection	Widespread PC transduction
	(2) Atxn1 Like/AAV serotype 2/1			Improved histology and behavioral profiles
Xia et al., 2004	siRNA/AAV2	B05 mice	Direct injection into the cerebellar lobules	Cerebellar morphology restoration, PC inclusions resolution, improved motor coordination
Keiser et al., 2014	RNAi/AAV serotype 2/5	SCA1 154Q/2Q knock-in mice	Deep cerebellar nuclei (DCN)	Preserved neurohistology and rotarod performance
Venkatraman et al., 2014	HDAC 3 depletion	(1) PC-specific HDAC3 null knock-in mice	N/A	(1) No improvement in cognitive and cerebellar function
		(2) SCA1 154Q/2Q mice (heterozygous HDAC3+/− mice)		(2) Deleterious effects both behaviorally and histologically

RNA interference (RNAi) has already been proven to be effective in a number of diseases caused by CAG repeat expansion. Harper et al. injected AAV2/1 targeting huntingtin (HTT) intracranially and found reduced HTT mRNA levels and protein expression in HTT transgenic mice. Mice showed improved behavioral outcomes and reduced neuropathic HTT abnormalities (Harper et al., 2005). Additionally, Monteys et al. found preferential silencing of the mutant HTT allele *in vitro* by miRNA. Preferential knockdown of the mutant allele was achieved *in vivo* when one of the miRNAs tested was delivered through AAV2 by striatal bilateral injection into HTT double transgenic mice, However, knockdown of the wild-type allele was also detected (Monteys et al., 2015). Miller et al. generated siRNA that silences the SCA 3-associated allele, ataxin-3 (ATXN3), targeting an adjacent single nucleotide polymorphism (SNP). *In vitro*, the RNAi approach successfully silenced both plasmid and viral expression of ATXN3 (Miller et al., 2003). *In vivo*, AAV2 vector was delivered via intracerebellar injection into SCA 3 mice. Rodriguez-Lebron et al. found that anti-ATXN3 miRNA suppressed ATXN3 expression and cleared nuclear accumulation of mutant ATXN3 (Rodriguez-Lebron et al., 2013). Lastly, for SCA 7, Ramachandran et al. found more than 50% reduction of mutant human and wildtype mouse ATXN7 allele after AAV2/1 subretinal space injection of miRNA into SCA 7 transgenic mice. Mice preserved normal retinal function after injection (Ramachandran et al., 2014). Keiser et al. found that ATXN1 levels can be reduced 30% by miS1 knockdown through AAV1 deep cerebellar nuclei injection in an adult rhesus. The intervention was well tolerated and there were no clinical complications. The biodistribution and tolerability of the miS1 treatment supports its use as a clinical therapeutic (Keiser et al., 2015; Table 3).

Xia et al. tested shRNA to target the *Sca 1* locus. They generated AAV serotype 2/1 (AAV 2/1) that expressed shRNA which was delivered via intracerebellar injection. This treatment resolved ATXN1 nuclear inclusions and PC morphology as well as improving motor coordination in transgenic B05 mice (Xia et al., 2004). Keiser et al. tested the therapeutic utility of SCA 1-targeted AAV 2/1 miRNA to suppress ATXN1 expression. The construct was delivered via deep cerebellar nuclei injection and resulted in PC transduction and improved gait and balance in transgenic B05 mice (Keiser et al., 2013). To our knowledge, siRNA or shRNA targeting ATXN1 has not been evaluated in 154Q/2Q mice. Keiser et al. found that miRNA therapy in 154Q/2Q mice was sufficient to reduce SCA 1 symptoms. They tested the efficacy of miRNA delivered via AAV 2/5 to the deep cerebellar nuclei. The RNAi inhibited SCA 1-related transcriptional changes, preserved the cerebellar lobule, and improved motor coordination (Keiser et al., 2014; Table 3).

The potential toxicity of small RNA should be noted. Grimm et al. studied the long-term effect of high shRNA expression in the livers of mice through AAV8 intravenous infusion. Of the 49 AAV/shRNA vectors tested, 36 caused does-dependent liver injury, with 23 of those leading to death. Grimm et al. found the morbidity to be associated with downregulation of miRNAs derived from the liver, possibly indicating competition with the shRNAs (Grimm et al., 2006). The study demonstrates the risk of oversaturating small RNA pathways with RNAi therapy.

As an alternate approach to RNAi, overexpression of the ATXN1L protein also represents a feasible therapy for SCA 1. ATXN1 and ATXN1L interact with a similar set of proteins, including CIC, through their ATXN1 and HMG-box protein 1 domain (AXH domain). Overexpression of ATXN1L could outcompete polyQ-ATXN1 for binding to CIC, reducing the amount of toxic complexes and disease severity (de Chiara et al., 2003; Lam et al., 2006; Bowman et al., 2007; Lim et al., 2008). Keiser et al. showed that overexpression of ATXN1L from an AAV2/1 vector has protective effects in B05 mice. In their study, human ATXN1L was expressed using AAV 2/1 and was delivered into the deep cerebellar nuclei. Mice receiving the injection exhibited improved gait, agility, and hind limb musculature and also decreased nuclear inclusions and PC loss, similar to that observed with the miRNA. Keiser et al. concludes that both overexpression of ATXN1L and RNAi are potentially viable treatment options for SCA 1 patients (Keiser et al., 2013; Table 3).

Alternatives to targeting the gene directly through RNAi and ATXN1L include histone deacetylase transcriptional repression. ATXN1 binds HDAC3, a class 1 histone deacetylase (HDAC) required for ATXN1 induced transcriptional repression (Karagianni and Wong, 2007). Venkatraman et al. tested the effect of PC-specific HDAC3 depletion in 154Q/2Q mice by siRNA knock down. However, mice did not show improvement in cerebellar or cognitive function. Additionally, Venkatraman et al. crossed a floxed HDAC3 mouse line with a mouse driving *Cre* expression from a *pcp-2* promoter. The *pcp-2* promoter turns on 6 days after birth in PC cells and reaches a maximum by 2–3 weeks after birth, which is about the time that transcriptional problems manifest (Lin et al., 2000; Gatchel et al., 2008). The offspring line has a conditional complete loss of HDAC3 in PCs and demonstrated dramatic PC degeneration and early onset ataxia. These results warn of the risk of neurotoxic side effects that can be caused by altering HDAC3 expression to treat SCA 1 (Venkatraman et al., 2014; Table 3).

In our opinion of the gene therapies, RNAi knockdown treats the cause of the disease while ATXN1L might not, as the polyQ-ATXN1 aggregates are still present. On the other hand, this cannot be definitive known until a side by side comparison between ATXN1L and RNAi with stringent control of the conditions is performed. Additionally, the issue of small RNA oversaturation arises. As with most gene therapies, the question also remains, is there adequate transduction of the therapeutic into the affected cells?

## Stem cell therapy

Stem-cell-based therapies represent a new strategy for spinocerebellar ataxias. Neurotransplantation has been performed in various cerebellar mutant mice using different types of cells and delivery techniques to stop PC degeneration and restore normal cerebellar architecture. Currently, a few studies have tried stem cell transplantation in SCA 1 mouse models and showed positive results both functionally and histologically.

An interesting approach by Chen et al. combines stem cell and gene therapy for gene delivery in SCA 1 mice. Bone marrow-derived cells (BMDCs) can fuse *in vivo* with somatic cells, including Purkinje neurons (Alvarez-Dolado et al., 2003; Weimann et al., 2003). Bone marrow derived cells (BMDC) were genetically modified using AAV7 to carry two SCA 1 modifier genes, DnaJB4 and Pcbp3. The genes were determined by Fernandez-Funez et al. to have attenuating effects on SCA 1 onset by assisting chaperone activity and transcription stabilization, respectively (Fernandez-Funez et al., 2000). Chen et al. delivered BMDC by a retroorbital sinus injection into 154Q/2Q mice. PC and BMDC fused to produce heterokaryons with PC properties. In the treated 154Q/2Q mice, there was some improvement in pathology. Mice showed a diminished number of nuclear inclusions and an increased number of surviving PC, highlighting the potential neuroprotective effects of this combined strategy (Chen et al., 2011; Table 4).

**Table 4:** **Overview of stem cell therapies for SCA 1**.

	**Mouse model**	**Type of stem cells**	**Route**	**Histology results**	**Functional results**
Chen et al., 2011	SCA1 154Q/2Q mice	BMDC (genetically modified using AAV7 to carry SCA 1 modifier genes)	Right retro-orbital sinus injection	Diminished nuclear inclusions, increased number of surviving PC	Not assessed
Chintawar et al., 2009	B05 transgenic mice	Subventricular zone derived NPCs	Stereotactic cerebellar white matter microinjection	Thicker molecular layer.	Improved motor skills
				Diminished PC loss	Normalized behavior
Matsuura et al., 2014	B05 transgenic mice	Bone marrow derived MSC	Intrathecal injections	Suppression of PC dendrites atrophy	Improved motor coordination
				Thicker molecular layer	

Chintawar et al. transplanted neural precursor cells (NPCs) from the subventricular zone of adult mice to cerebellar white matter of B05 mice. Mice received injections into three separate sites of the cerebellar white matter at 5-, 13-, or 24-weeks-old time points where cerebellar pathology is not yet observed, PC loss begins, and PC are mostly abnormal, respectively. Grafted NPCs only migrated to the cerebellar cortex in 24-week-old mice. Additionally, motor skills only improved in mice treated at 24 weeks when compared to shams. When grafted NPCs did migrate to the cerebellar cortex, grafts did not adopt PC characteristics, but NPC-grafted B05 mice did exhibit thicker molecular layer and diminished PC loss (Chintawar et al., 2009; Table 4).

Matsuura et al. found that intrathecal injection of mesenchymal stem cells (MSCs) into the meningeal covering of the cerebellum improved PC organization in B05 mice injected at 5 weeks of age. At 24 weeks of age, untreated B05 mice displayed multilayered PC as a result of ectopically located PC bodies, but mice of the same age injected with MSCs had monolayered PCs. Additionally, MSC treatment reduced PC dendrite atrophy and normalized behavior and motor deficits (Matsuura et al., 2014; Table 4).

Stem cell therapies on non-SCA 1 mouse models are relevant to mention. In Lercher mutant mice, a mouse model characterized by the selective early post-natal death of PCs in the cerebellum, Jones et al. found that bone marrow-derived mesenchymal stem cells injected into the cerebellum migrated throughout the cerebellum. Their results showed an increase in the number of surviving PCs due to the neurotrophic factors released by adjacent grafted cells as well as an improvement in motor function (Jones et al., 2010).

Stem cell therapy in SCA 2 mice has also proven successful. Human MSC were delivered intravenously at 12, 23, 33, and 42 weeks of age (disease onset for untreated mice was 33–40 weeks of age). The injections resulted in a delay in the onset of motor function deterioration. In the same study, human MSC were injected into the cerebellum through the foramen magnum both before and after motor function loss but, unlike the intravenous route, there was no improvement (Chang et al., 2011).

Two clinical trials have looked at the safety and efficacy of stem cell transplantation in SCA. In a phase I/II trial, Jin et al. intrathecally and intravenously infused human umbilical cord mesenchymal stem cells (UC-MSC) into 16 patients with genetically diagnosed SCA 1, 2, or 3. Results showed that no serious transplantation side effects occurred during the 12 month follow-up period. Berg Balance Scale (BBS) and International Cooperative Ataxia Rating Scale (ICARS) scores improved for at least 6 months following the transplantation. Results were scored by a blinded neurologist (Jin et al., 2013) (NCT01360164).

Dongmei et al. also studied the effect of intrathecal injection of umbilical cord mesenchymal stem cells (UC-MSC) in 14 patients with SCA. The types of SCA were not specified. Serial weekly injection, with four injections total, significantly improved Activity of Daily Living Scale (ADL), and ICARS scores. ICARS and ADL scores decreased significantly 1 month after treatment. For follow-up, 8 patients remained stable for an average period of 9 month and 6 progressed after treatment with average stabilization of 4 months (Dongmei et al., 2011).

These studies may represent a proof of principle of the therapeutic potential of stem cells in SCA. However, both trials were open label, and substantial placebo effects cannot be discounted. Further clinical studies including more patients need to be performed in order to better assess safety and efficacy.

## Alternative therapies

While gene and stem cell therapies are currently most prominent, many alternative therapies, including protein delivery, are also potentially relevant treatments for SCA 1. Main approaches include vascular endothelial growth factor (VEGF), cAMP-dependent protein kinase A (PKA) inhibitory polypeptide, and 3,4-diaminopyridine.

Cvetanovic et al. found that vascular endothelial growth factor (VEGF) improves the SCA 1 phenotype in 154Q/2Q mice. VEGF is an angiogenic and neurotrophic factor, and it is down-regulated in SCA 1 mice. Cvetanovic et al. crossed 154Q/2Q mice with transgenic mice that overexpress VEGF. These mice exhibited thicker molecular layers and improved motor performance. This study went further to show the positive effect that recombinant VEGF has on 154Q/2Q mice. As it cannot cross the blood brain barrier (BBB), an intracerebroventricular osmotic pump was inserted subcutaneously into 11-week-old mice to deliver VEGF via a catheter into the right lateral ventricle over 2 weeks. Mice with the pump exhibited thicker molecular layers and improved motor performance. The authors proposed that VEGF could be a biomarker for human disease progression (Cvetanovic et al., 2011; Table 5).

**Table 5:** **Overview of other therapies for SCA 1**.

	**Treatment**	**Animal model**	**Route**	**Outcome**
Cvetanovic et al., 2011	VEGF	154Q/2Q mice	Pharmacologic: intraventricular infusion	Improved pathological hallmarks and motor function
			Transgenic: gene over-expression	
Hearst et al., 2014a	PKA inhibitory polypeptide (Synb1-ELP-PK)	B05 mice	Intraperitoneal/intranasal	Decreased intranucelar inclusions, improved PC morphology
Hourez et al., 2011	3,4-diaminopyridine	B05 mice	Subcutaneous injection	Normalized PC firing rate, reduced PC atrophy, improved motor function
Hearst et al., 2014b	Focused Laser Light Hyperthermia	B05 mice	Cerebellar	Hsp70 production, suppressed PC loss, improved motor function
Watase et al., 2002	Lithium	154Q/2Q	Dietary	Improved motor coordination, learning, and memory. Increased Dendritic branching in hippocampal pyramidal neurons
Perroud et al., 2013	Lithium	154/2Q	Dietary	Increased metabolic processes, specifically higher purine levels
Iizuka et al., 2015	Memantine	154Q/2Q	Dietary	Attenuated PC loss and vagus motor neuron loss. Extended life span and reduced weight loss

PC degeneration in SCA 1 is enhanced by the phosphorylation of S776 of polyQ-ATXN1 by PKA (Chen et al., 2003; Emamian et al., 2003; Jorgensen et al., 2009). Hearst et al. (2014a) engineered a PKA inhibitory polypeptide to prevent the formation of nuclear inclusions. The PKA inhibitory polypeptide (Synb1-ELP-PKI) is composed of a cell-penetrating peptide (Synb1), a heat responsive elastin-like peptide (ELP) carrier to increase peptide half-life, and a PKA inhibitory peptide (PKI). Using B05 mice, Synb1-ELP-PKI was delivered through the intraperitoneal or intranasal injection route. Both routes allowed the peptide to successfully cross the BBB and localize in the cerebellum. Mice showed decreased intranuclear inclusions and improved PC morphology. Motor function was not assessed (Hearst et al., 2014a; Table 5).

Hourez et al. (2011) found that aminopyridines might have positive neuroprotective and symptomatic effects for SCA 1. 3,4-diaminopyridine is an organic compound that blocks potassium channels and is used to treat neuronal dysfunction (Kirsch and Narahashi, 1978). Before SCA 1 symptoms present, B05 mice show reduced PC firing rate. Acute subcutaneous injection of 3,4-diaminopyridine in young B05 mice normalized the firing rate of PCs and improved motor function. Chronic subcutaneous injection, partially prevented PC atrophy. This effect was associated with increased brain-derived neurotrophic factor (BDNF), suggesting that protection against atrophy is possibly carried out by BDNF, which occurs secondary to the return of electrical activity (Hourez et al., 2011; Table 5). Adding to the argument that aminopyridines increase PC firing frequency, (Alviña and Khodakhah, 2010) reported that 4-aminopyridine restored pacemaking precision in the PCs in a mouse model of episodic ataxia 2 (Alviña and Khodakhah, 2010). A clinical trial of 4-aminopyridine (dalfampiridine) was completed in 2014. In a crossover study 20 patients with either SCA 1, 2, 3, or 6 received an oral dose of 4-aminopyridine for 4 weeks and 4 weeks of placebo with a 2 week washout period in between. One patient had adverse effects to the drug. Results showed that 4-aminopyridine did not significantly improve performance on the Timed 25 Feet Walking Test, the Scale of Assessment and Rating of Ataxia (SARA), or the Biomechanical Assessment of Gait–Stride Length (NCT01811706).

Other alternatives strategies that are less invasive, including whole body vibration (WBV), heat shock protein (HSP) stimulation, and oral doses of lithium, have also shown promising results and could be used alone or as an adjunct to other treatments. Kaut et al. investigated the effect of stochastic whole body vibration on SCA 1, 2, 3, and 6 patients in a double blind sham controlled study. WBV stimulates the neuromuscular system through vibration, and it has already shown to improve balance and mobility in patients with Parkinson's (Haas et al., 2006). WBV was applied on 4 sequent days, the treatment consisting of five stimulus trains of 60 s duration at a frequency of 6.5 Hz and 60 s resting time between stimuli. The sham group received the same treatment with a frequency of 1 Hz. Improvements in gait, posture, and speed of speech were seen but no response in limb kinetics and ataxia of speech was observed (Kaut et al., 2014). However, it is necessary to mention a Huang et al. (2014) review of WBV trials. The review states that there is insufficient evidence to support or negate if WBV can reduce symptoms in SCA among other diseases claiming more trials need to be performed with better methodological and reporting quality.

Hearst et al. (2014b) based on the neuroprotective role of chaperone HSPs known to modulate polyglutamine protein aggregation, explored the effects of focused laser light induced hyperthermia (HT) on HSP-mediated protection against ATXN1 toxicity in both cell culture model and transgenic mice. This study revealed that mild cerebellar HT stimulated the production of Hsp70 to a significant level and markedly suppressed the SCA 1 phenotype and PC loss as compared to sham-treated control animals (Hearst et al., 2014b). The nuclear inclusions in PCs stain positive for Hsp70 and Hsp40, ubiquitin and proteasomal subunits. Over expression of HDJ-2, an Hsp40 chaperone reduces protein aggregation (Cummings et al., 1998). Cummings et al. went on to find that overexpression of Hsp70 chaperone suppresses neuropathy and improves motor function in B05 mice (Cummings et al., 2001).

Lithium treatment has the potential to reduce SCA 1 symptoms in humans. A Watase et al. study showed that dietary lithium treatment resulted in improved motor coordination as well as learning and memory in 154Q/2Q mice. At 10 weeks of age, motor improvement was noted both in mice that received the treatment pre-symptomatically and post-symptomatically. Lithium treatment increased dendritic branching in mutant hippocampal pyramidal neurons (Watase et al., 2007). Perroud et al. also found that lithium treatment improved the motor coordination of 154Q/2Q mice. Mice receiving lithium exhibited increased metabolic processes, particularly higher purine levels. Perroud et al. propose that purine metabolites might have a neuroprotective effect (Perroud et al., 2013). Lithium is involved in a large number of cell processes; it is not clear what combination is alleviating the symptoms. A human phase I trial of oral lithium was completed in 2010; results have yet to be published (NCT00683943).

Memantine, a low-affinity non-competitive N-methyl-d-aspartate (NMDA) receptor antagonist is a possible drug. Iizuka et al. orally administered memantine to 154Q/2Q mice from 4 weeks old to death. Mice receiving the treatment lived longer and did not lose as much weight as typical 154Q/2Q mice. Mice receiving memantine had attenuated PC loss as well as motor neuron loss in the dorsal motor nucleus of the vagus. The study claims that these results exhibit that activation of extrasynaptic NMDA receptors lead to neuronal cell death in 154Q/2Q mice (Iizuka et al., 2015). However, the study fails to show the effect of memantine on SCA 1 symptoms is through its role as an NMDA antagonist. Memantine also affects the dopamine (Seeman et al., 2008), serotonin (Rammes et al., 2001), and acetyl choline receptors (Aracava et al., 2005) which would complicate its use as a SCA 1 therapy. Memantine has potential as a SCA 1 therapeutic but the mechanism of action needs to be better understood.

Glial cells have not received as much attention as neuronal pathology in SCA 1 studies, despite reports of gliosis in the brains of deceased SCA 1 patients (Genis et al., 1995; Gilman et al., 1996). In many neurological diseases, such as Amyotrophic Lateral Sclerosis and Parkinson's disease, astrocyte and microglia activation changes, including proinflammatory cytokine release, can reduce neuronal survival (Glass et al., 2010). Cvetanovic et al. observed hypertrophy of Bergmann glia, a sub-type of astrocyte that is located near Purkinje neurons, in 154Q/2Q mice using Glial Fibrillary Acidic Protein (GFAP). Hypertrophy was noticed in the presymptomatic period of the 154Q/2Q model at 8 weeks old, and the GFAP staining increased with disease progression. Cvetanovic et al. also observed hypertrophy of microglia cells in 154Q/2Q mice by staining for ionized calcium-binding adapter molecule 1 (Iba1). Like the Bergmann glia, microglia hypertrophy began at 8 weeks of age and increased with disease progression. Additionally, Cvetanovic et al. found glial activation occurs presymptomatically in B05 mice where only Purkinje neurons express ATXN1. Pro-inflammatory cytokine micro RNA levels increased alongside glial levels as well (Cvetanovic et al., 2015). Cvetanovic has recently received funding from the National Ataxia Foundation to test PLX3397, a drug that removes microglia from the brain without adverse effects in mice under laboratory conditions (Elmore et al., 2014), as a therapeutic.

## Treatments used in other SCA types

Two treatments currently being tested in other forms of SCA are worthy of mention, specifically Rhophylax, an IgG, and Dantrolene, a ryanodine-receptor inhibitor. Both therapies have the potential to be tested on SCA 1 as well.

It is believed that inflammation may contribute to neuronal dysfunction, although the pathophysiology is not known. Evert et al. (2001) found upregulation of mRNAs encoding metalloproteinase 2 (MMP-2), an endopeptidase matrix, the cytokine stromal cell-derived factor 1α (SDF1α), the transmembrane protein amyloid precursor protein, and the interleukin-1 receptor-related Fos-inducible transcript in SCA 3 mice. Immunohistochemical analysis of human SCA 3 brain tissue found increased expression of MMP-2 and amyloid β-protein (Aβ) in pontine neurons containing nuclear inclusions. Additionally, increased numbers of astrocytes and microglial cells were found in the pons of SCA 3 patients (Evert et al., 2001). In light of these results, a clinical trial is being initiated to administer intravenous immune globin (IVIG)- Rhophylax, an IgG, to SCA 1, 2, 3, 6, 10, and 11 patients to observe how the drug effects SCA symptoms as well as nerve and motor function (NCT02287064). In the pilot for the clinical trial, three patients received three courses of IVIG-Rhophylax at 2 grams/ kilogram body weight over 5 days. Courses were 4 weeks apart. The patients in the pilot had SCA 3, SCA 5, or sporadic SCA. Results showed a 40% reduction in SARA total score and 10–20% average improvement in gait parameters. Patients improved the best after the third course and continued to improve until 28 days after the last infusion. Patients improvements declined by 56 days after the last infusion (Zesiewicz et al., 2014).

Calcium-mediated neurodegeneration has attracted considerable attention in the past couple of decades (Jimenez-Jimenez et al., 1996; Wojda et al., 2008); therefore a discussion on calcium-focused treatments for SCA 1 is warranted. Studies have shown SCA 1 model exhibiting down-regulation of proteins involved in PC calcium homeostasis, particularly Calbindin and Paravalbumin (Vig et al., 2000, 2001). Dantrolene has been tested on SCA 2 and SCA 3. Liu et al. found that mutant ataxin-2 facilitated inositol phosphate-induced Ca^2+^ release in cultured PC of a transgenic SCA 2 mouse model. Dantrolene blocked the Ca^2+^ release *in vitro* and, when given orally to mutant transgenic mice from 2 to 11 months old substantially reduced expression of SCA 2 (Liu et al., 2009). Chen et al. demonstrated that mutant ATXN3 associated with type 1 inositol 1,4,5-triphosphate receptor, an intracellular calcium release channel. They went on to demonstrate oral administration of dantrolene to SCA 3 transgenic mice stopped neuronal cell loss and improved motor performance, and stated dantrolene should be considered as a possible therapeutic for SCA 3 patients (Chen et al., 2008). Dantrolene should also be considered as a potential therapeutic for SCA 1.

## Hurdles

At first glance, gene and cell therapies for SCA 1 seem to be straightforward. Gene therapy approaches involve the delivery of a transgene that can replace, silence, or inhibit the defective gene. Alternatively, the delivery of stem cells might replace the dying cell pool or provide a protective environment that would prevent these cells from dying. But in practice the process is more complex and a certain number of variables need to be controlled and optimized.

First, a route of delivery needs to be chosen. While peripheral administration (such as intravenous, intramuscular, or intranasal) sounds more appealing and less invasive, it raises several barriers. Higher dosages are needed to reach the same effect compared to a central delivery approach. Transduction of non-CNS tissues carries the risk of off-target effects. Finally, most gene therapy vectors lack the ability to cross the blood brain barrier.

Direct delivery to the CNS can be achieved via intraparenchymal injections to brain and spinal cord structures or via injections into the CSF (intrathecal or intraventricular). While these approaches offers the benefit of being more targeted, for SCA 1 a specific brain region may need to be chosen, such as deep cerebellar nuclei or cerebellar hemispheres. Additionally, surgical techniques need to be developed and optimized to be more reproducible and accurate. While these methods are more invasive, direct delivery of viral vectors and stem cells into the CNS have proven safe and effective (Marks et al., 2010; Gutierrez et al., 2015).

Concerning stem cells, it is also very important to determine the factors behind successful grafts. Many studies do not characterize graft survival or the factors that allow these cells to slow the rate of disease progression. The description of graft characteristics and features would help predict the outcomes and help choose the optimal cell type for type of injection and brain region receiving the injection.

## Conclusion

A wide variety of therapies for SCA 1 are being investigated to preserve PCs and slow disease onset and progression. While stem cell therapies are the ones that have currently reached the clinical trial stage, recent progress in gene and protein delivery appear to be equally promising for SCA 1 treatment. Mouse models, particularly the 154Q/2Q mouse, provide a valuable model of the disease. However, the mechanism of action of polyQ-ATXN1 is not entirely understood and further research is needed to understand the disease process. Conceptually speaking, RNAi therapies, be they siRNA, shRNA, or miRNA-based, seem to be the most promising, since they deal directly with the root cause of the disease. All three RNAi options have been efficacious in SCA 1 models as well as models for other polyQ diseases. However, transducing a large majority of neurons throughout the brain remains a hurdle for gene therapy. Clinical trials will be necessary to determine which therapies or combinations of therapies will result in the best outcomes for patients.

## Author contributions

JW is the primary author of the review. DO and AD edited the review and provided constructive criticism and approval. NB is an editor of the review and provided final approval.

### Conflict of interest statement

NB is a paid consultant for Agilis, MRI Interventions, Voyager, Oxford Biomedica, Q Therapeutics and Neuralstem Inc. He is a founder of Switch Bio Holdings and former employee of Above and Beyond LLC up to December 1st 2015. The other authors declare that the research was conducted in the absence of any commercial or financial relationships that could be construed as a potential conflict of interest.
